# Comments on Stone et al, “A case of secondary syphilis presenting like pemphigus with positive direct immunofluorescence”

**DOI:** 10.1016/j.jdcr.2024.07.039

**Published:** 2024-08-27

**Authors:** Camila Cordero Pacheco, Shivani Sinha, Brett Sloan, Diane Whitaker-Worth, Michael J. Murphy

**Affiliations:** a University of Puerto Rico School of Medicine, San Juan, Puerto Rico; b Department of Dermatology, University of Connecticut Health Center, Farmington, Connecticut

**Keywords:** desmoglein, direct immunofluorescence, infectious diseases, pemphigus, plasma cell, syphilis

*To the Editor:* Syphilis is recognized for its diverse manifestations. In response to 2 recent articles addressing rare pemphigus-like presentations of syphilis, we wish to raise awareness of such cases considering a comparable case observed in our clinic.

In the article “A case of secondary syphilis presenting like pemphigus with direct immunofluorescence,” Stone et al^1^ present a middle-aged man with eroded, crusted, erythematous papules, and plaques on the head, neck, and trunk. Although this presentation may resemble other pemphigus variants, further workup demonstrated a positive direct immunofluorescence consistent with pemphigus vulgaris, the presence of *Treponema pallidum* on immunostaining, and a positive rapid plasma reagin, ultimately leading to the diagnosis of lues maligna-type secondary syphilis. The authors compared this case with a previously published article, “A pemphigus-like presentation of secondary syphilis,” in which Kopelman et al^2^ documented a case of a teenage boy presenting with scattered eroded, erythematous, scaly plaques, vesicles and bullae, and subtle purpuric macules on the palms and soles. Although rapid plasma reagin was positive in this case, direct immunofluorescence and enzyme-linked immunosorbent assay for IgG against serum desmoglein-1 and desmoglein-3 were negative. In comparing these cases, the clinical variability and complexity of diagnosing syphilis is highlighted.

The purpose of this letter was to contribute another case of a pemphigus-like presentation of syphilis observed in our clinic. A 52-year-old woman presented with a 9-month eruption involving her face, upper extremities, and trunk consisting of annular, scaly, and violaceous plaques with crusting and ulcerations (Fig 1). She reported associated fevers and vesicles on the skin in the initial stages of this eruption. Laboratory work-up revealed positive IgG desmoglein-1 and borderline/indeterminate desmoglein-3 antibody levels, and a punch biopsy revealed lichenoid and perivascular dermatitis with numerous plasma cells (Fig 2). In light of these findings, a rapid plasma reagin titer was drawn, resulting in a positive value at 1:512. However, immunostaining did not reveal spirochetes. The patient was treated with weekly intramuscular penicillin G for 3 weeks, which led to improvement of her skin lesions with remnant postinflammatory hyperpigmentation.

**Fig 1:**
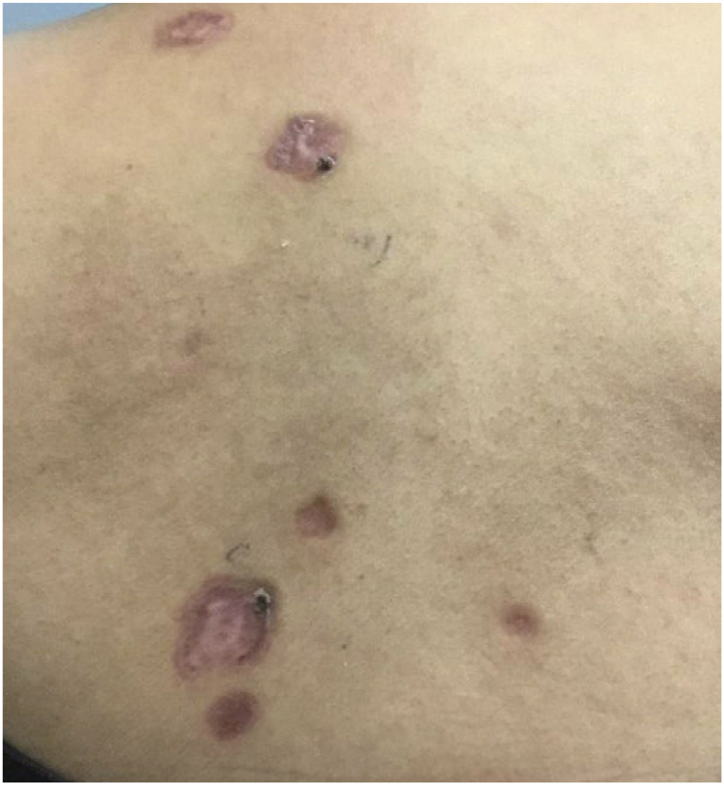
Cutaneous syphilis manifestations: violaceous to brown, mottled-appearing plaques with scalloped borders on the back.

**Fig 2:**
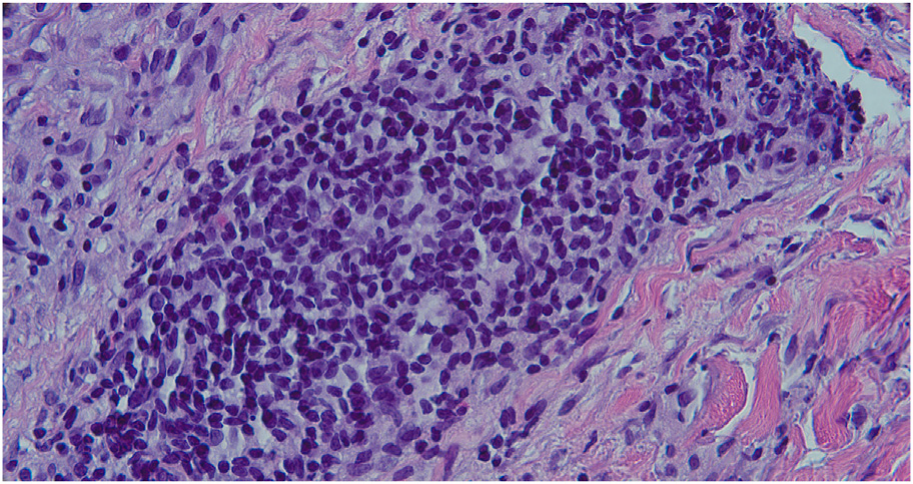
Numerous plasma cells on biopsy. (Hematoxylin-eosin stain; original magnification: ×400.)

These 3 cases highlight the clinical variations of syphilis presenting with pemphigus-like findings. “Syphilitic pemphigus,” another coined term for a bullous variant of syphilis, has been previously reported in infected newborns presenting with pustules and bullae involving the palms and soles.^3^ Histopathology of these cases featured acantholysis and eosinophils, consistent with the 2 reports highlighted above, however did not report positive direct immunofluorescence.^3^ In addition to a variation of histopathologic features, our case suggests that the inability to detect *T pallidum* through immunostaining does not exclude diagnosis given its limited sensitivity.^4^ To our knowledge, our patient is the first reported case with a positive desmoglein-1, adding to the increasing number of reports of pemphigus-like syphilis with varying diagnostic results. With this is in mind, we would like to emphasize the importance considering a pemphigus-like syphilitic variant in the differential diagnosis of eroded, nonspecific lesions without other identifiable causes given timely treatment is imperative to mitigate long-term complications of this infectious disease.

## Conflicts of interest

None disclosed.
